# Clinical and Genetic Findings of the First Report of PAPA Syndrome in Brazil

**DOI:** 10.1155/2021/6660937

**Published:** 2021-12-13

**Authors:** Sérgio Júlio Fernandes, Maria Isabel Valdomir Nadaf, Nauro Hudson Monteiro, Izabel Nazira Nadaf, Cleiton Ribeiro Lélis, Bianca Yumi Takano, Bárbarah Gabriella de Camargo Monteiro, Nyvea Gabriella de Camargo Monteiro, Olga Akiko Takano, Leonardo Oliveira Mendonça

**Affiliations:** ^1^Hospital e Pronto Socorro Municipal da Secretaria Municipal de Saúde de Cuiabá, Cuiabá, Brazil; ^2^Department of Pediatrics, Universidade Federal de Mato Grosso, Cuiabá, Brazil; ^3^Universidade de Cuiabá, Cuiabá, Brazil; ^4^Division of Clinical Immunology and Allergy, Universidade de São Paulo School of Medicine, Universidade de São Paulo, São Paulo, Brazil; ^5^Laboratory for Immunological Investigation (LIM-19), Heart Institute, University of São Paulo, São Paulo, Brazil; ^6^Center for Rare and Immunological Disorders, DASA-Hospital 9 de Julho, São Paulo, Brazil

## Abstract

**Background:**

PAPA syndrome (MIM #604416) is a rare monogenic autoinflammatory disease genetically transmitted in an autosomal dominant trait that results from missense mutations in the proline-serine-threonine phosphatase-interactive protein 1 (PSTPIP1) gene located on chromosome 15 and is characterized by sterile pyogenic arthritis, pyoderma gangrenosum, and cystic acne. We describe the clinical and molecular findings of two related Brazilian patients with PAPA syndrome. *Case Presentation*. A 7-year-and-3-month-old boy with nonconsanguineous parents had had recurrent pyoarthritis since the age of 5 years and 8 months. During his last and long hospitalization, the lack of improvement with antibiotics, evidence of increased inflammatory activity, repeated arthrotomies, draining purulent fluid that had negative cultures, and the history of trauma, all on in a clinical background of pyoarthritis, led to the suspicion of an autoinflammatory syndrome. This was confirmed by the good clinical response to corticotherapy. Genetic sequencing confirmed the diagnosis of PAPA syndrome, with the pathogenic mutation c.688 G > *A* (p. Ala230Thr) in the PSTPIP1 gene present in the patient and in the mother.

**Conclusions:**

This case illustrates that in children with recurrent/recalcitrant sterile recurrent pyogenic arthritis/osteomyelitis, the possibility of an underlying immunological condition should be considered. In both, recurrent infections or recurrent inflammation, many genes involved in the inborn errors of immunity can be associated, and a correct and precocious diagnosis is necessary to avoid mobility and mortality. To the best of our knowledge, this is the first report of PAPA syndrome in Brazil.

## 1. Introduction

PAPA syndrome is a rare autoinflammatory disease (MIM #604416) clinically characterized by the syndromic forms condensed in the acronym: pyogenic arthritis, pyoderma gangrenosum, and cystic acne [1]. Genetically, heterozygous mutations, mainly of missense origin, along the PSTPIP1 (a.k.a. CD2BP1) gene are transmitted in an autosomal dominant fashion and are associated to PAPA syndrome. This gene encodes the protein PSTPIP1, a cytoskeletal protein within hematopoietic cells that serves as a support for the binding of other cellular proteins, such as pyrin, tyrosine phosphatase, c-Abl, CD2, and Wiskott–Aldrich syndrome protein (WASP) [2]. Through these interactions, PSTPIP1 regulates several cellular functions, including interleukin-1*β* (IL-1*β*) release, cytoskeletal organization, cell migration, and T cell activation [3]. However, as many patients mimicking PAPA syndrome lack the genetic finding, high levels of serum IL-18 seem to differentiate those who carry genetically pathogenic mutations in the PSTPIP1 gene [4].

Here, we describe for the first time two related patients with PAPA syndrome in the Central-West region of Brazil. Besides the case report, we also emphasize the necessity of disease awareness for autoinflammatory syndromes in the pediatric age group.

## 2. Case Presentation

The patient is a 7-year-old boy born and raised in the Central-West region of Brazil (Cuiabá) from nonconsanguineous parents. He exhibited odontogenic abscesses associated with unexplained sinusitis at age 4 with resolution of the condition after standard treatment. At the age of 5 years, a low-impact trauma in the right elbow, evolved to a local disproportional inflammation within a few hours, was treated with immobilization with plaster splint. Over the following two weeks, the patient developed fever and persistent, painful edema diagnosed as pyoarthritis in the right elbow. At that time, laboratory analysis revealed mild anemia (hemoglobin (Hb) = 10.0 g/dL), hematocrit (Ht) = 30.5%), high levels of acute reactant markers (erythrocyte sedimentation rate (ESR) = 21 mm, and C-reactive protein (CRP) = 15.3 mg/dL (reference value (RV) < 1 mg/dL)). Also, a computed tomography (CT) of the right elbow revealed the presence of diffuse periosteal reactions affecting the proximal metaphyseal regions of the radius and ulna, as well as of the distal metaphyseal region of the humerus plus voluminous joint effusion and diffuse soft tissue elbow enlargement, especially in the medial aspect. With the diagnostic impression of probable sepsis of the elbow, the patient underwent arthrotomy with surgical drainage of a large amount of purulent fluid with lumps, and broad-spectrum antibiotic therapy was initiated. After 72 hours of antibiotic therapy and arthrotomy, the patient maintained the marked painful edema in his right elbow and culture of the synovial fluid came out negative. The patient remained hospitalized until the 53rd postoperative day when he presented with sudden and pronounced edema and pain in the left knee after trauma caused by falling from his own height while playing in the corridor of the ward. He underwent to another arthrotomy of the left knee with discharge of abundant purulent liquid. Due to persistent painful edema, the patient underwent another arthrotomy of the left knee for drainage of a joint effusion that was repeatedly purulent, all with negative cultures.

**Figure 1:**
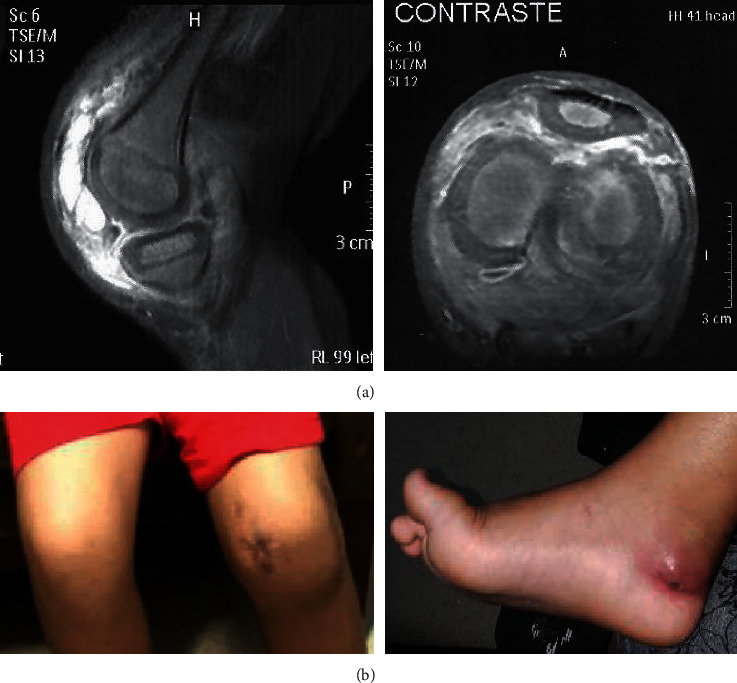
(a) Magnetic resonance imaging of the left knee. Sagittal plane (left); transverse plane (right), T2-weighted contrast. Presence of an extraarticular infiltrative process with involvement of the subcutaneous tissue and epidermis; inflammatory infiltration of adjacent muscle tissue, without evident collections; small foci of inflammatory edema affecting the subcortical bone marrow; extensive synovitis in the patellar recess and intraarticular involvement. In the transverse plane, there is an increase in the inflammatory process, with volumetric impingement of the popliteus neurovascular bundle. (b) Clinical characteristics of the pyoarthritis. Sequelae with increased left knee volume (left); the inflammatory process in the right ankle caused by trauma from the buckle on footwear (right).

Of note, a magnetic resonance imaging of the left knee (Figure 1(a)) showed significant inflammatory arthropathy, with joint effusion associated with diffuse synovitis; extension of the inflammatory process into the periarticular soft parts affecting the popliteus and quadriceps muscle; extrusion of the medial meniscus; and small foci of inflammatory edema affecting the subcortical bone marrow of the trochlear sulcus and the medial femoral condyle, which may have corresponded, according to the radiological report, to the focus of incipient osteomyelitis.

After 60th day of hospitalization, the patient was discharged, but two weeks after, there was another need for hospitalization due to another arthritis. At that time, laboratory analysis demonstrated high levels of acute reactants markers, and he never evaluated to sepsis. Another arthrotomy was necessary again with pus drainage.

Because of the recalcitrant situation and the suspicion of an inborn error *o* immunity, a target gene panel (Invitae panel, 407 genes, Jeffrey Modell Foundation Partnership Program) was requested, and an already reported mutation in the PSTPIP1 (c.688 G > *A* (p. Ala230Thr)) gene was found in the patient and in the mother.

Curiously, just after genetic founding, the mother discovered similar cases in the family and reported of herself having a prolonged episode of arthritis in the right knee, which began after trauma during a soccer game at the beginning of her adult life. During the reported occasion, she fully recovered, and other members of the family have not yet been genetically investigated. She also reported of having had cystic acne throughout adolescence and young adulthood, with nowadays facial skin marked by scar depression. Finally, genetic sequencing was not just essential for the diagnosis but also for genetic counseling and clarification of the underlying condition. During 2 years of follow-up, no episodes of pyoderma gangrenosum were noted, neither in the mother nor in the index patient.

After being discharged from his last hospital stay, the patient presented at the age of 8 with two more subsequently episodes of left knee arthritis triggered by low-impact local trauma and a new episode of arthritis, this one in the right ankle, due to trauma secondary to the use of inadequate footwear (Figure 1(b)). In these episodes, the response to treatment with prednisone 1 mg/kg over 3 weeks was satisfactory, followed by gradual withdrawal. However, no specific measure could be accessed to infer the impact of the nonpreventive treatment with anti-IL1, as suggested in the literature. Unfortunately, anti-IL1 blockers are not available, in both public and private systems in Brazil, and that is the reason the patient did not receive it.

## 3. Discussion

Here, we describe for the first time two related patients with PAPA syndrome from Brazil, both harboring the A230 T heterozygous mutation in the PSTPIP1 gene. PAPA syndrome is a very rare autoinflammatory condition, and clinical approach to diagnosis is challenging due to its heterogeneous manifestations. Moreover, there are no specific radiographic findings or biomarker of PAPA what turns final diagnosis even trickier. Genetic sequencing is essential for the final diagnosis, and up to date, at least 66 variants were reported in PSTPIP1 gene (https://infevers.umai-montpellier.fr/web/search.php?n=5) and just 8 are classified as pathogenic or likely pathogenic [5].

Due to heterogeneous presentation, delays in diagnosis and diagnostic errors in inborn errors of immunity (IEI) are common [3]. Specially in PAPA syndrome, pyogenic arthritis is the most common and usually the first clinical finding of the syndrome. Pyoderma gangrenosum is the least common occurrence. Cystic acne is more frequent in puberty and can last for years into adulthood [6]. Furthermore, trauma events triggering pyoarthritis episodes are known as the pathergy phenomenon, which is a characteristic of PAPA syndrome [7]. Taking into account all these findings, as presented in our patient, inborn errors of immunity and PAPA syndrome should be suspected in patients with recurrent pyogenic arthritis or recurrent osteomyelitis. Although acne and pyoderma gangrenosum are absent from his clinical picture, the patient is still a child and may manifest in adolescence or adulthood.

There is no specific bone/articular radiological finding in PAPA syndrome, and usually, voluminous joint effusion is the main finding. Articular drainage is essential in such cases to rule out infection and confirm the sterile underlying process. However, 30–50% of cases of septic arthritis in children are not detected by culture [8, 9]. Thus, we emphasize that children with recurrent episodes of sterile or infective arthritis must be immunologically and genetically evaluated in order to rule out an underlying IEI. In addition, signs of osteolytic lesions and bone alterations mimicking osteomyelitis, as presented in our patient, have already been described in PAPA syndrome, but is not specific of the syndrome [10, 11].

Genetic sequencing is essential for the diagnosis, therapeutic management, and avoidance of new untreatable sequelae in PAPA syndrome. All pathogenic mutations related to the classical phenotype of PAPA are located in the coiled-coil domain of the PSTPIP1 gene (amino acid residues 123–288) [5, 12]. Curiously, two specific mutations in the same gene and domain (E250 K and E257 K) are associated to a different and specific condition, the PAMI syndrome, what requires caution in genotype versus phenotype correlation. PAPA but not PAMI has a great response to anti-IL1 therapies, and bone marrow transplantation has not yet been reported in PAPA patients [13]. Moreover, some other nonspecific clinical forms of pyoderma gangrenosum were linked to mutations in other domains of the gene and yet without a clear immunological relation.

In conclusion, the early diagnosis of PAPA syndrome by genetic sequencing in this case avoided new and repeated hospitalizations in search of a bacterial etiology of pyoarthritis, wasteful collection of samples for laboratory tests, invasive surgical drainage and arthrotomy procedures, and excessive and unnecessary use of antibiotics. Although expensive and not covered by the Brazilian Unified Health System, genetic sequencing was permitted in this patient, an accurate diagnosis and thus the correct treatment (only anti-inflammatory drugs). Therefore, in similar cases, it is necessary for orthopedists, infectologists, and clinicians to consider the possibility of PAPA syndrome when faced with recurrent pyogenic arthritis without an explicit infectious etiology but associated with trauma.

## Data Availability

The data used to support the findings of this study are collected from the medical record of the Hospital *e* Pronto Socorro Municipal da Secretaria Municipal de Saúde de Cuiabá.
